# No Evidence of Microsatellite Instability in Head and Neck Squamous Cell Carcinoma of Non‐Smokers and Non‐Drinkers

**DOI:** 10.1111/jop.70120

**Published:** 2026-01-19

**Authors:** F. J. Mulder, T. F. B. Gielgens, E. J. de Ruiter, R. de Bree, M. F. C. M. van den Hout, B. Kremer, S. M. Willems, E. J. M. Speel

**Affiliations:** ^1^ Department of Otorhinolaryngology and Head & Neck Surgery GROW‐Research Institute for Oncology and Reproduction, Maastricht University Medical Center Maastricht the Netherlands; ^2^ Department of Otorhinolaryngology Amsterdam University Medical Center Amsterdam the Netherlands; ^3^ Department of Pathology University Medical Center Utrecht Utrecht the Netherlands; ^4^ Department of Head and Neck Surgical Oncology University Medical Center Utrecht Utrecht the Netherlands; ^5^ Department of Pathology GROW‐Research Institute for Oncology and Reproduction, Maastricht University Medical Center Maastricht the Netherlands; ^6^ Department of Otorhinolaryngology and Head & Neck Surgery Erasmus Medical Center Rotterdam the Netherlands; ^7^ Department of Pathology and Medical Biology University Medical Center Groningen Groningen the Netherlands; ^8^ Department of Pathology and Clinical Bioinformatics Erasmus Medical Center Rotterdam the Netherlands

**Keywords:** head and neck cancer, immune checkpoint inhibitors, immunohistochemistry, microsatellite instability, non‐drinkers, non‐smokers, polymerase chain reaction

## Abstract

**Introduction:**

While the prevalence of microsatellite instability (MSI) is low in the whole head and neck squamous cell carcinoma (HNSCC) population, it has been suggested to be more prominent in tumors of non‐smokers. Therefore, the goal of this study was to determine the presence of MSI in a cohort of well‐defined HNSCC of non‐smokers and non‐drinkers (NSND).

**Methods:**

Clinical characteristics and tumor tissue of 119 NSND with HNSCC were retrospectively collected and analyzed for MLH1, PMS2, MSH2, and MSH6 protein expression on tissue microarrays (TMA). In case of negative staining for one of these mismatch repair proteins in the TMA cores, immunohistochemistry (IHC) was repeated on a whole slide section and additional molecular analyses were performed using polymerase chain reaction (PCR) and quantitative PCR (qPCR).

**Results:**

Two cases showed dubious loss of MSH2 expression, one of these with concurrent dubious loss of MSH6 on the TMA. However, MSH2 and MSH6 expression was retained on whole slide sections and PCR and qPCR analyses did not show any mutations, compatible with a microsatellite stable result.

**Conclusion:**

This study shows no single case with MSI in the NSND subgroup of HNSCC. Although a deficient DNA mismatch repair system is a predictive biomarker for response to immune checkpoint inhibitors, we found no evidence to support routine analysis of MSI in HNSCC, also not in the subgroup of NSND.

## 1 Introduction

In recent years, tumor immune checkpoint inhibitors (ICIs) are being increasingly used in cancer treatment. It is a therapeutic approach in which these ICIs block proteins that normally restrain immune cells, including PD‐1, PD‐L1, or CTLA‐4, thereby unleashing T cells to recognize and attack cancer cells more effectively. Because only a select group of cancer patients benefit from immunotherapy with ICIs, numerous studies have been carried out to identify biomarkers that may predict patient response [1] One of the predictive biomarkers of ICI therapy response is a deficient DNA mismatch repair (MMR) system [2] The MMR system is key for recognizing and repairing base mismatches and misbindings during DNA replication [1] Cancers harboring a deficient MMR are often hypermutated, leading to microsatellite instability (MSI) by accumulating mutations in monomorphic microsatellites that are prone to mismatch errors [2, 3] In clinical settings, immunohistochemical analysis of MMR proteins is routinely performed to detect MSI in multiple cancers [4] Alternatively, polymerase chain reaction (PCR)‐based microsatellite testing and next‐generation sequencing (NGS) approaches may be used for this purpose.

The anti‐PD‐1 monoclonal antibody pembrolizumab has been approved by the Food and Drug Administration for the treatment of patients with unresectable or metastatic solid tumors who have been identified as having deficient MMR or being MSI‐high (MSI‐H) [5]. Although widely variable incidences of MSI‐H have been reported in head and neck squamous cell carcinoma (HNSCC), exome‐sequencing data analysis from The Cancer Genome Atlas suggests it is around 1.2% [4, 6]. Therefore, the Society for Immunotherapy of Cancer (SITC) recommends that MSI should currently not routinely be tested as part of HNSCC treatment protocols [7]. While the prevalence of MSI is low in the whole HNSCC population, it has been suggested to be more prominent in tumors of non‐smokers [8, 9]. Therefore, the goal of this study was to determine the presence of MSI in a cohort of well‐defined HNSCC of non‐smokers and non‐drinkers (NSND).

## 2 Materials and Methods

### 2.1 Patients

Consecutive patients with HNSCC were selected at two University Medical Centers in the Netherlands, as described previously [10]. Inclusion criteria were: ≥ 18‐years‐old NSND patients with HNSCC, available formalin‐fixated, paraffin‐embedded (FFPE) tumor tissue, and > 3 years follow up. Non‐smoking was defined as having no history of smoking, non‐drinking as having no history of alcohol consumption (not even “sporadic” alcohol consumption), as reported in the patients' medical records during both their first presentation at the Head and Neck outpatient clinic, and during the pre‐anesthesia screening before panendoscopy or surgical resection. Patients with a second primary tumor in the head and neck region, tumors outside the upper aerodigestive tract, or a cervical metastasis of unknown origin were excluded. Human papillomavirus and Epstein‐Bar virus status has been determined before with HPV‐positivity in all 10 oropharyngeal tumors, one oral squamous cell carcinoma (OSCC) of the alveolar process, and a nasopharyngeal tumor [10]. The other three nasopharyngeal tumors were EBV‐positive.

The Medical Ethics Review Committee of the Maastricht University Medical Center (2018–0573) has approved this study and the principles outlined in the Declaration of Helsinki were followed. All data and tissues were handled according to General Data Protection Regulation.

### 2.2 Tissue Microarrays and Immunohistochemistry

FFPE blocks were retrieved from the departments of Pathology (MPTC PA1711‐path‐114) and hematoxylin and eosin sections were digitally evaluated with a senior head and neck pathologist (SW or MH) using the Pannoramic viewer (3DHISTEC, Budapest, Hungary). Per patient, three 0.6 mm tumor tissue cores and one normal epithelium core were selected and placed in three tissue microarrays (TMAs).

Five μm FFPE TMA sections were subjected to immunohistochemistry (IHC) for MMR proteins MLH1 (clone ES05), PMS2 (clone EP51), MSH2 (clone FE11), and MSH6 (clone EP49 (Dako Omnis—Agilent Technologies, Carpinteria, CA)). Standardized immunostainings were performed on a Dako Omnis autostainer using the EnVision FLEX+ Mouse (LINKER) kit according to the manufacturer's protocol. Normal colon tissue was used as positive control. Cases were deemed microsatellite stable when all four IHC markers showed any nuclear positivity in the tumor tissue. In case tumor nuclei were negative or showed very weak (dubious) staining for at least one of the MMR proteins in the TMA cores (as assessed by consensus between FM and MH), IHC was repeated on a whole slide section and additional molecular analyses were performed.

For these molecular analyses, DNA was isolated from five μm FFPE whole slide sections with at least 30% tumor cells (indicated by MH) using the Maxwell DNA FFPE kit (Promega Corporation, Madison, Wisconsin, USA). MSI testing was performed by PCR amplification of the monomorphic microsatellite markers BAT25, BAT26, CAT25, NR21, NR22, and NR24. Additionally, quantitative PCR (qPCR) was performed with the fully automated Idylla MSI Test (Biocartis, Mechelen, Belgium), analyzing mutations in MSI loci ACVR2A, BTBD7, DIDO1, MRE11, RYR3, SEC31A, and SULF2 according to the manufacturer's instructions.

## 3 Results

**FIGURE 1:**
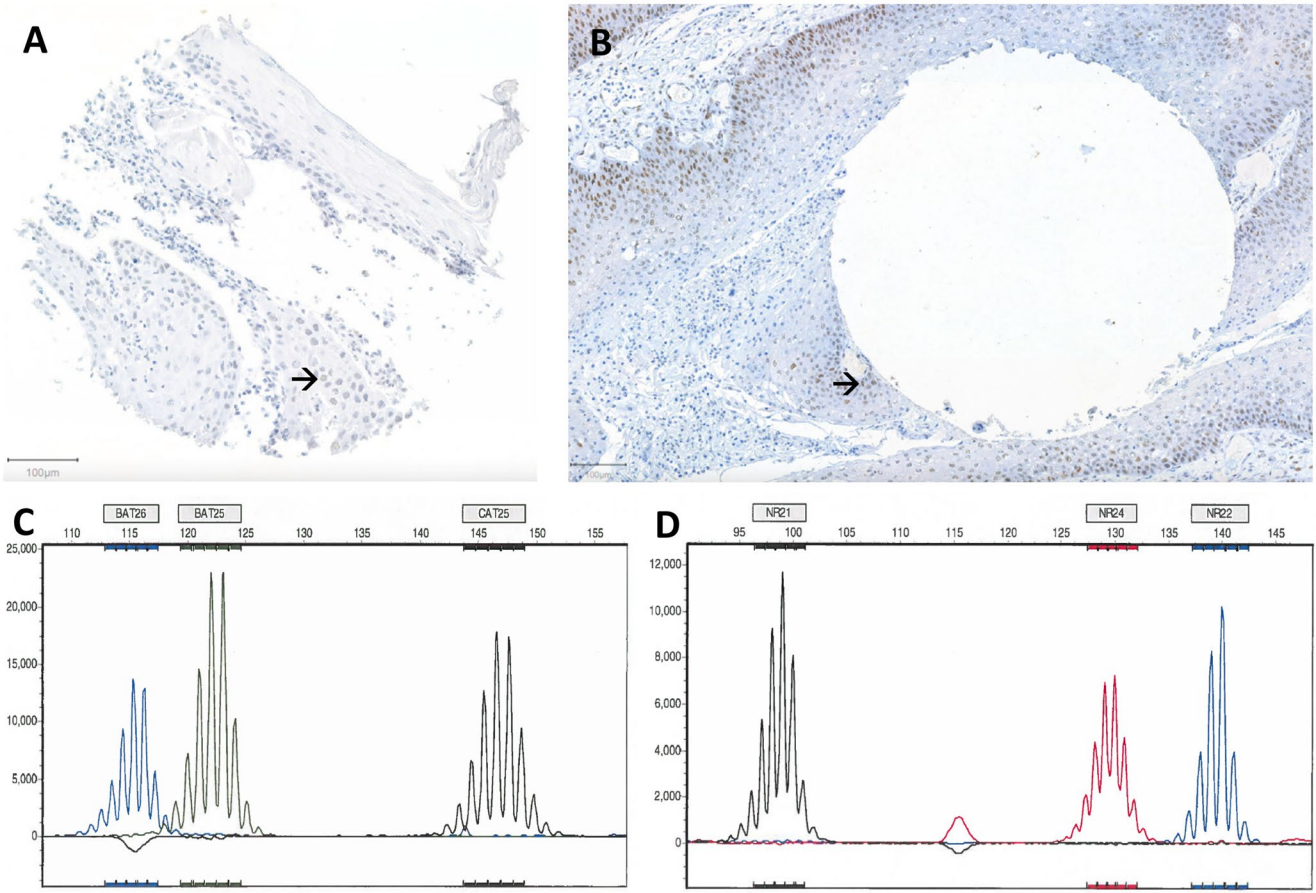
(A) Example of a tissue microarray core of one of the two tumors with dubious nuclear positivity for MSH2 staining (example marked with ➔). (B) However, the tumor cells surrounding the area where the tissue microarray core was taken from on the whole slide section did show clear nuclear MSH2 staining (➔). Images were digitally evaluated at 200× magnification as shown; an area of 100 μm is marked in both images. (C, D) Microsatellite PCR analysis for monomorphic microsatellite markers BAT25, BAT26, CAT25, NR21, NR22, and NR24 on genomic DNA of this tumor did not show repeats length alteration, fitting a microsatellite stable result.

Immunohistochemistry for MMR proteins was analyzed in 119 cases (Figure 1, Figure S1; Table 1). Two cases showed dubious loss of MSH2 expression: a pT1N0M0 lateral border of the oral tongue tumor and a pT4N0M0 tumor of the maxilla, the latter with concurrent MSH6 loss on the TMA. However, whole slide sections of tumor tissue showed no immunohistochemical loss of any of the MMR proteins (Figure 1). Additional PCR and qPCR analyses on the tumors of these two patients did not show any mutations, compatible with a microsatellite stable result (Figure 1).

**TABLE 1:** Clinical characteristics of head and neck squamous cell carcinoma in non‐smokers and non‐drinkers.

Clinical characteristics		Total (*n* = 119)
Age in years	Median (interquartile range)	74.9 (14.6)
		***n* (%)**
Sex	Female	93 (78)
	Male	26 (22)
Location	Hypopharynx	1 (0.8)
	Larynx	9 (7.6)
	Nasopharynx^a^	4 (3.4)
	Oropharynx^b^	10 (8.4)
	*Oral cavity*	
	Alveolar process^c^	24 (20)
	Buccal mucosa	5 (4.2)
	Floor of mouth	13 (11)
	Hard palate	1 (0.8)
	Lip	5 (4.2)
	Oral tongue	41 (35)
	Retromolar triangle	6 (5.0)
T‐stage	1	33 (28)
	2	26 (30)
	3	13 (11)
	4	37 (31)
N‐stage	0	78 (66)
	1	18 (15)
	2	21 (18)
	3	2 (1.7)
M‐stage	0	114 (96)
	1	5 (4.2)
Recurrence	Yes	31 (26)
	No	88 (74)
HPV	Positive	12 (10)
	Negative	107 (90)

^a^ 3 EBV positive and 1 HPV positive.

^b^ All HPV positive.

^c^ 1 HPV positive.

## 4 Discussion

This study did not find evidence of MSI in a large cohort of HNSCC in NSND. This was in line with the review of Amaral‐Silva et al. about MMR proteins in malignant oral lesions (of predominantly smokers). When applying the current IHC standard (MSI in case of a complete loss of cell nuclei expression for a MMR protein), only the most recent of the included studies showed a patient with no MSH2 expression (1/113 OSCC of that study, smoking history of this patient not reported) [2, 6]. All other studies showed either positive staining or, in some cases, a lower expression (but no lack of expression) for MLH1, PMS2, MSH2, and MSH6 [6]. These results contradict the conclusion of Field et al. [9], who suggested that MSI is a mechanism of carcinogenesis in non‐smokers. However, they assessed a different set of microsatellite markers via PCR than the current European Society for Medical Oncology (ESMO) standard panel of poly‐A mononucleotide repeats used in the current study, which could explain the difference in conclusions [2].

For tumors with MSI, including those caused by the Lynch syndrome, the ESMO suggests IHC using the four MMR proteins as the first method of MSI testing for its wide availability [2]. In case of indeterminate IHC results, MSI‐PCR molecular testing is indicated, as was done in the current study. Because of the low MSI‐H prevalence in HNSCC, the SITC recommends against standard MSI testing (e.g., via NGS analysis) unless the patient is already having a genome profile performed providing such information [7]. Yet, since MSI subtypes in OSCC are susceptible to immunotherapeutic methods, Arslan Bozdag et al. [11] propose in their recent review on MSI in OSCC that it is advisable to consider MSI testing in specific cases like recurrent or metastatic HNSCC, for whom ICIs would be a viable treatment option.

One of the limitations of this study was that some of the FFPE material had > 25 years of storage, resulting in slight differences in staining intensity between the tumors with a longer and shorter storage time. However, the TMA blocks were freshly sectioned before IHC, all positive controls were adequate, and in case of dubious/weakly positive staining additional analyses were performed. Secondly, this study used TMAs which only comprise part of the original tumor. Nevertheless, three cores were taken per patient's tumor to take into account heterogeneity within the tumor. Lastly, few of the included HNSCC were outside the oral cavity, so the conclusion that tumors of the hypopharynx, larynx, oropharynx, and nasopharynx do not show MSI is less firm. Yet, HNSCC of NSND are typically located in the oral cavity or oropharynx, so this distribution of tumor sub sites was to be expected.

## 5 Conclusion

While MSI has been suggested to be more prominent in HNSCC of non‐smokers, this study shows no single case with MSI in the subgroup of NSND. Although a deficient MMR is a predictive biomarker for ICI response, these results strengthen the advice of the SITC to not routinely check for MSI in HNSCC, also not in the subgroup of NSND.

## Funding

The authors have nothing to report.

## Conflicts of Interest

B. Kremer and E.J.M. Speel report a relationship with the Maastricht University Medical Center that includes: research funding grants from Pfizer and Novartis. S.M. Willems reports a relationship with the University Medical Center Groningen that includes: research funding grants from Roche, Pfizer, MSD, Lilly, AstraZeneca, and Bayer.

## Supporting information


**Figure S1:** Examples of tissue micro array cores with H&E staining (A, B), and staining for MLH1 (C, D), PMS2 (E, F), MSH2 (G, H), and MSH6 (I, J). Each column represents a single patient. H showed dubious nuclear positivity for MSH2 (the same core as presented in Figure 1), all other cores (C–J) show nuclear positivity for the mismatch repair proteins.

## Data Availability

The data that support the findings of this study are available on request from the corresponding author. The data are not publicly available due to privacy or ethical restrictions.
