# Supplementary material for: No Evidence of Microsatellite Instability in Head and Neck Squamous Cell Carcinoma of Non‐Smokers and Non‐Drinkers
Source: J Oral Pathol Med. 2026 Jan 19;55(5):611–5. doi: 10.1111/jop.70120 (PMC13149765; doi:10.1111/jop.70120)
Supplement: Supplementary file 1 — Figure S1: Examples of tissue micro array cores with H&E staining (A, B), and staining for MLH1 (C, D), PMS2 (E, F), MSH2 (G, H), and MSH6 (I, J). Each column represents a single patient. H showed dubious nuclear positivity for MSH2 (the same core as presented in Figure 1), all other cores (C–J) show nuclear positivity for the mismatch repair proteins. [file JOP-55-611-s001.docx]

**A**

**C**

**E**

**G**

**I**

**B**

**D**

**F**

**H**

**J**


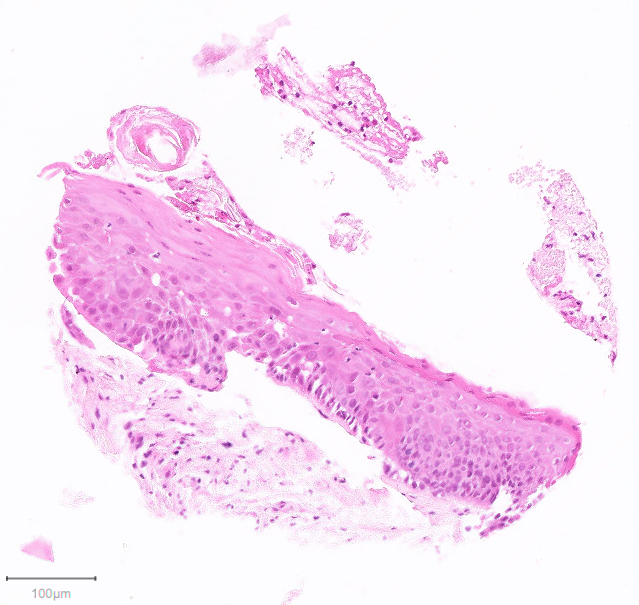

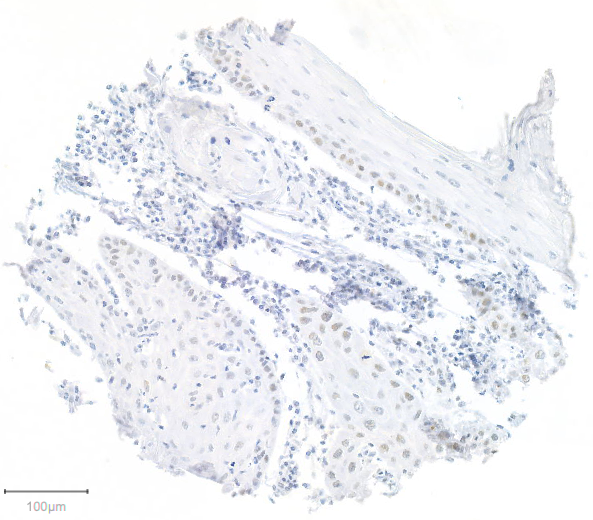

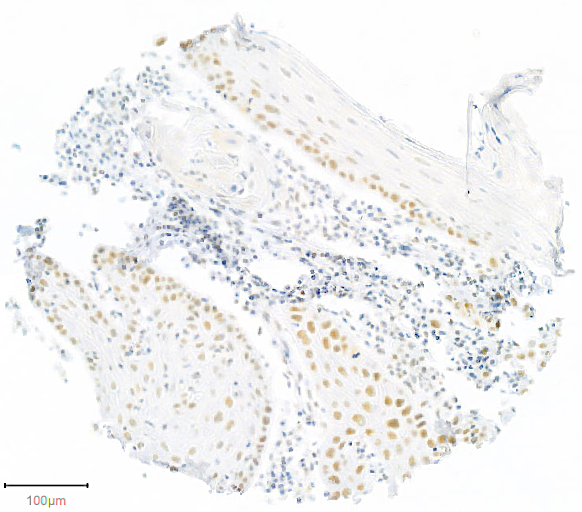

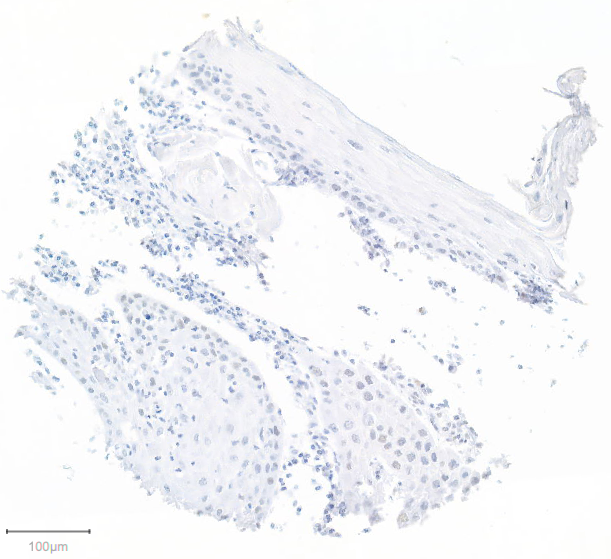

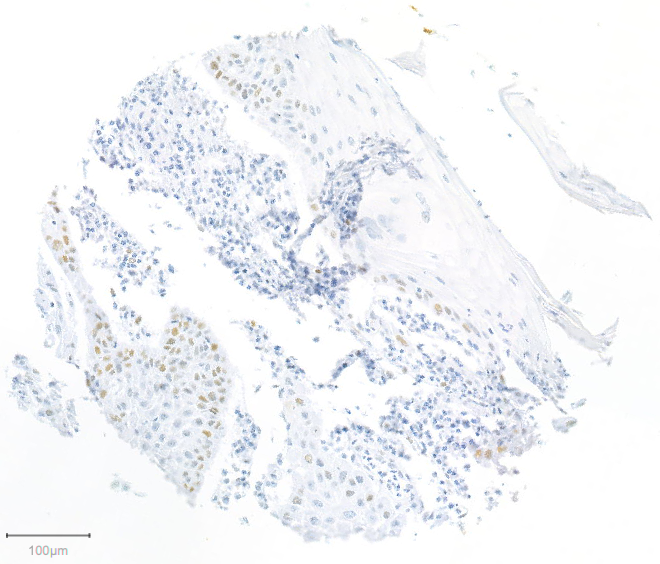

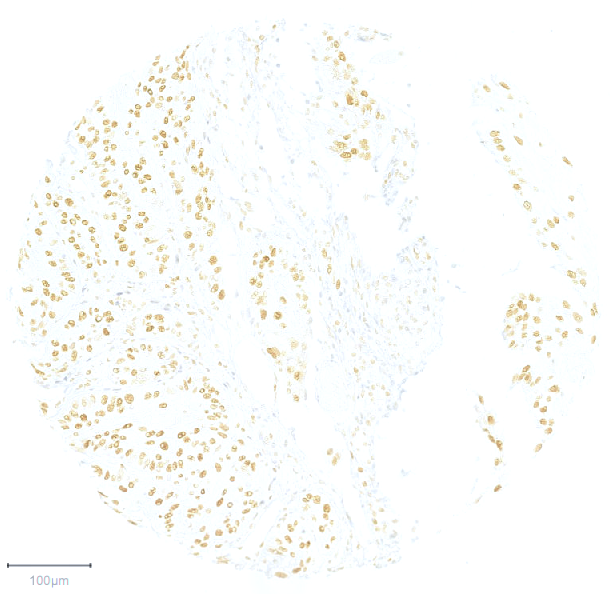

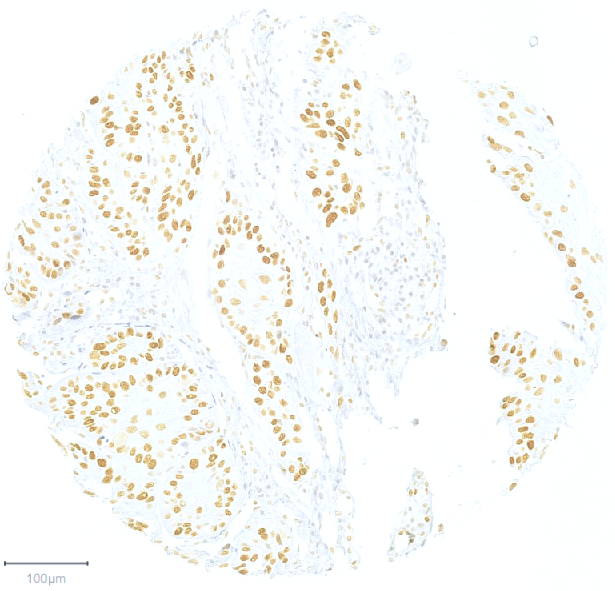

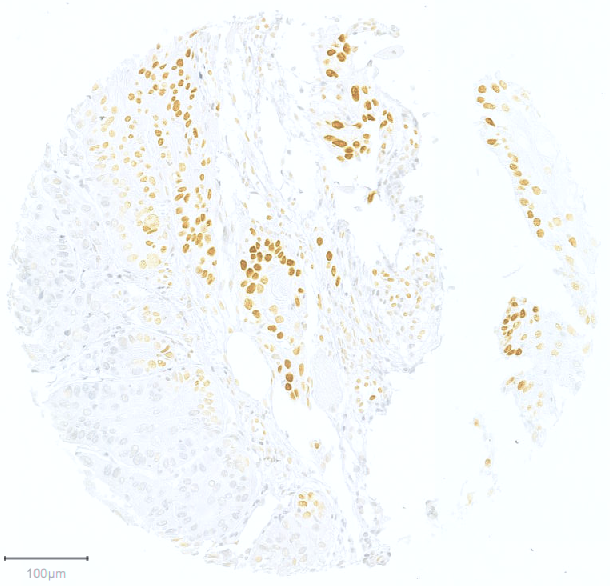

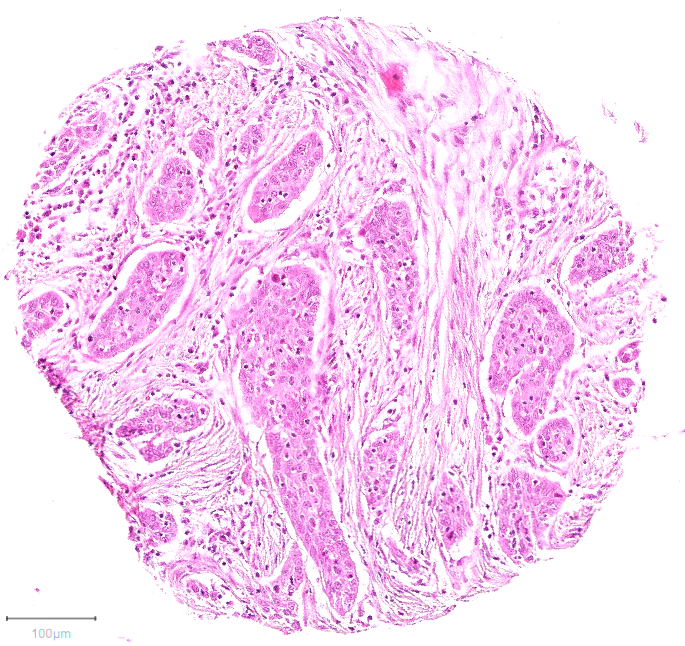

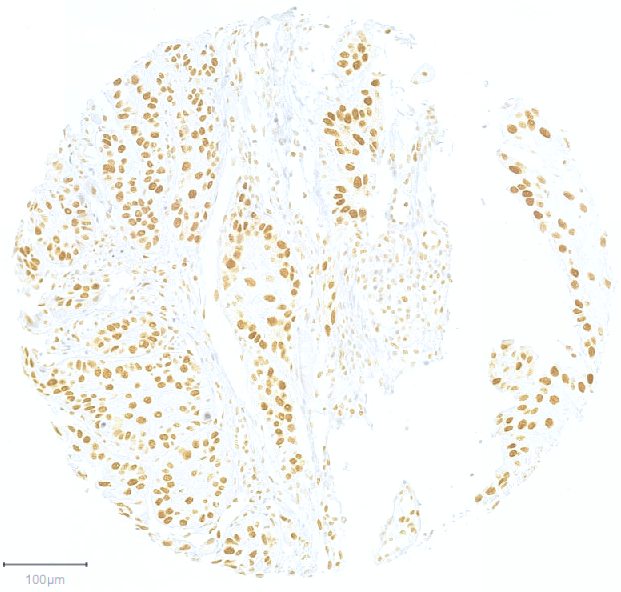


**Supplementary figure 1**. Examples of tissue micro array cores with H&E staining (**A, B**), and staining for MLH1 (**C, D**), PMS2 (**E, F**), MSH2 (**G, H**), and MSH6 (**I, J**). Each column represents a single patient. **H** showed dubious nuclear positivity for MSH2 (the same core as presented in Figure 1), all other cores (**C – J**) show nuclear positivity for the mismatch repair proteins.
